# TNF as Biomarker for Rapid Quantification of Active Staphylococcus Enterotoxin A in Food

**DOI:** 10.3390/s120505978

**Published:** 2012-05-10

**Authors:** Reuven Rasooly, Bradley Hernlem

**Affiliations:** Western Regional Research Center, Agricultural Research Service, US Department of Agriculture, Albany, CA 94710, USA; E-Mail: bradley.hernlem@ars.usda.gov

**Keywords:** enterotoxin, food poisoning, TNF, immunomagnetic beads

## Abstract

*Staphylococcus aureus* is a major bacterial pathogen which causes clinical infections and food poisoning. This bacterium produces a group of twenty-one enterotoxins (SEs). These enterotoxins have two separate but related biological activities. They cause gastroenteritis and function as superantigens that activate large numbers of T cells. The current method for detection of enterotoxins activity is an *in vivo* monkey or kitten bioassay; however, this method is not practical to test on a large number of samples. Several immunological assays have been developed however, but these assays cannot distinguish between active toxin which causes food poisoning and inactive toxin, which can bind antibody, but shows no toxicity. The current study demonstrates that short term *ex vivo* exposure of primary naïve CD4^+^ T-cells or splenocytes to SEA induces differential expression and secretion of tumor necrosis factor (TNF) protein. We used immunomagnetic beads coated with anti-SEA antibody to specifically isolate SEA from food. After the eluted toxin was added to the cells SEA biological activity was measured by quantifying TNF protein expression or secretion.

## 1. Introduction

*Staphylococcus aureus* is one of the major bacterial pathogens causing clinical infections and foodborne illnesses [1]. This bacterium produces about 50 virulence factors that have a wide range of biological activities [2], including a group of twenty-one known enterotoxins (SEs) that act at very low concentrations and have two different, but related, biological activities; emetic activity and superantigenic activity [3–5]. SEs act on the gastrointestinal tract, and as a superantigen (SAg) on the immune system. Unlike normal antigens, SAg can bind to MHC class II molecules, which are expressed on the surface of a subtype of CD4^+^ T cells [6,7], which perform the role of both antigen presenting cell (APC) and T cell receptor (TCR). Therefore CD4+ T cells are able to present SAg to themselves or to neighboring CD4^+^ T cells without a safety mechanism which requires interaction between two types of cells [7]. This stimulates ∼20% of the naïve T-cell population which all share particular sequences within the variable region of the β chain (V-β) of the T cell receptor (TCR) [8]. The current test to detect active SEs is an *in vivo* monkey or kitten bioassay [9,10]. This procedure has low sensitivity and poor reproducibility and requires many experimental animals. Immunological assays have been developed for several SEs, but these methods cannot distinguish between active and inactive toxin. Recent contamination with SEA causing an extensive outbreak with 13,420 cases in Japan [11,12], emphasizes the need to develop better methods to detect active SEs. In this work we used three complimentary approaches for measuring tumor necrosis factor (TNF). Differential expression of TNF was secreted in large quantities following short term *ex vivo* exposure to SEA therefore; TNF can be used as a biomarker for early detection of biologically active SEA in food.

## 2. Experimental Section

### 2.1. Splenocyte Isolation

Spleen cells from C57BL/6 female mice were prepared aseptically and disrupted using a syringe and needle in Russ-10 cell culture medium [made by combining 450 mL of RPMI 1,640 medium without glutamine (Gibco, Carlsbad, CA, USA), 50 mL Fetal bovine serum (Hyclone, Logan, UT, USA), 5 mL 200 mM glutamine (Gibco), 5 mL antibiotic-antimycotic (Gibco—containing penicillin, streptomycin, and fungizone), 5 mL non-essential amino acid mix (Gibco), 5 mL sodium pyruvate (Gibco), and 0.25 ml of 100 mM β-mercaptoethanol (Sigma, St. Louis, MO, USA). Cells were centrifuged at 200 × g at 4 °C for 10 min. Red blood cells were then lysed by adding red cell lysis buffer (0.15 M NH_4_Cl, 10 mM KHCO_3_, 0.1 mM Na_2_EDTA). Cells were again centrifuged and resuspended in Russ-10 medium, and viable cells were counted using trypan blue and a hemocytometer.

### 2.2. Splenocyte Proliferation Assay

Cells were placed in 96-well plates (1 × 10^6^/mL, 0.2 mL) in Russ-10 medium and treated with various concentrations of SEA followed by incubation at 37 °C in a 5% CO_2_ incubator. After incubation at various time points, cell proliferation was measured by adding 5-bromo-2-deoxyuridine (BrdU)-labeled DNA to each well 4 h before fixation as described by manufacturer's instructions (Calbiochem, San Diego, CA, USA). Spectroscopic measurements were made of optical density at 620 nm and 450 nm.

### 2.3. Positive Isolation of Murine CD4^+^ T cells

Murine CD4^+^ T cells were isolated using a positive selection (Dynabeads Mouse CD4 L3T4), according to the manufacturer's instructions. Briefly, Splenocytes were resuspended in isolation buffer (PBS supplemented with 0.1% BSA and 2 mM EDTA) at a concentration of 1 × 10^7^/mL and incubated with washed Dynabeads (25 μL of Dynabeads per 10^7^ cells) for 20 min on ice with gentle rotation. After incubation, cells and Dynabeads were placed on a magnet for 2 min. The supernatant was removed and the bead-bound cells were washed 3 times with isolation buffer. The bead-bound cells were resuspended in RUSS-10 media (10^7^ cells per 100 μL of media) and DETACHaBEAD mouse CD4 was added (10 μL per 10^7^ cells) and incubated for 45 min with gentle rotation at room temperature. The detached beads were washed 3 times and resuspended in media.

### 2.4. Coating Magnetic Beads with Anti-SEA Antibody

One hundred μL of Dynabeads M-280 tosylactivated (Invitrogen, Carlsbad, CA, USA) were washed twice with 600 μL of 0.1 M sodium borate buffer, pH 9.5, and diluted in the same buffer to 2 × 10^9^ beads/mL. Purified anti-SEA antibody (30 μg) was added to 1 × 10^8^ beads (50 μL). The antibody and beads were incubated for 24 h at 37 °C on a slow shaker to facilitate covalent binding. The coated beads were washed twice for 5 min at 4 °C with 1 mL phosphate-buffered saline (PBS), pH 7.4, containing 0.1% bovine serum albumin (BSA), washed once for 4 h at 37 °C with 0.2 M Tris-HCl, pH 8.5, containing 0.1% BSA, and washed once more for 5 min at 4 °C with PBS, pH 7.4, containing 0.1% BSA. The beads were resuspended in 50 μL of the Tris-BSA buffer.

### 2.5. Sample Preparation of Food

Our preliminary result suggested that the food matrices themselves are somewhat cytotoxic, thus interferes with analytical outcomes. To overcome this obstacle the food were diluted; 20 g of each preserved chicken or green beans peas (Gerber) were added to 20 mL of PBS. Then the foods were spiked with 5 μg/mL, 1 μg/mL, 200 ng/mL, 5 ng/mL or 1 ng/mL of SEA.

### 2.6. Sample Binding and Disassociation of SEA from Beads

Fifteen μL of the immunomagnetic beads were incubated with a tilting motion at 4 °C with 1 mL of spiked green bean or chicken. After 24 h, the tube was placed on a magnet for 2 min to collect the beads. The beads were washed twice with PBS, pH 7.4, containing 0.1% BSA. Toxin was eluted with 7.5 μL of 100 mM glycine HCl (pH 2.5) then neutralized with 7.5 μL of 2X tris buffered saline (TBS) (pH 8.3).

### 2.7. Measurement of TNF by Cytometric Bead Array

TNF concentration in cellular supernatant was assayed using Mouse Cytometric Bead kit (BD Biosciences, San Diego, CA, USA), following manufacturer's protocol.

### 2.8. Measurement of Intracellular TNF Protein Levels

Cells were fixed and permeabilized following the procedure of BD kit Cat # 555028. Intracellular staining of TNF with an APC anti-TNF antibody was performed following manufacture's protocol (BD Pharmingen, San Diego, CA, USA).

### 2.9. Measurement of TNF by ELISA

TNF concentration in cellular supernatant was measured using BD OptEIA mouse TNF ELISA Set (BD Biosciences), following manufacture's protocol.

## 3. Results

### 3.1. Measuring Biologically Active SEA in Food by Splenocyte Proliferation Assay

**Figure 1.**
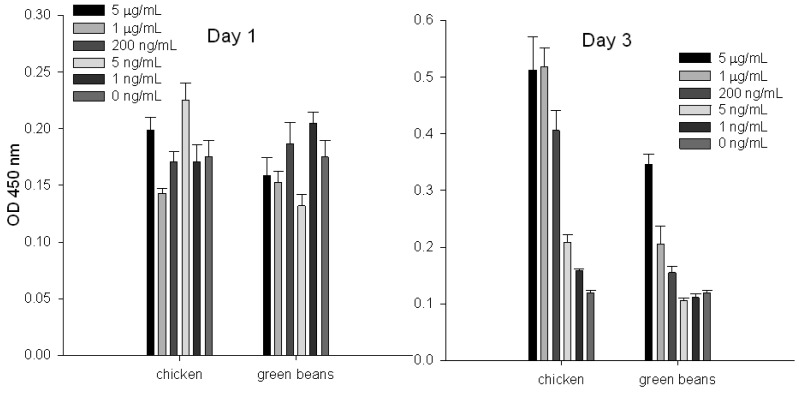
Detection and quantify of SEA in food using BrdU cell proliferation assay. Chicken and green beans were spiked with increasing concentrations of SEA. Immunomagnetic beads were used to extract the toxin. Eluted toxin from beads was incubated with splenocytes. Proliferation was measured by incorporation of BrdU in newly synthesized DNA. Error bars represent standard error (N = 7).

To evaluate the ability of the splenocyte proliferation assay to quantify specific SEs in chicken and green beans, we spiked these food products with increasing concentrations of SEA and then extracted the toxin by using immunomagnetic beads coated with anti-SEA antibody. Our result (Figure 1) shows that by measurement of incorporated BrdU into cellular DNA, toxin cannot be detected at day 1; it can be detected at day 3. We also noticed that at day 3 there is a correlation between eluted SEA concentrations and cell proliferation.

### 3.2. Intracellular TNF Protein Expression for Detection of Biologically Active SEA

**Figure 2.**
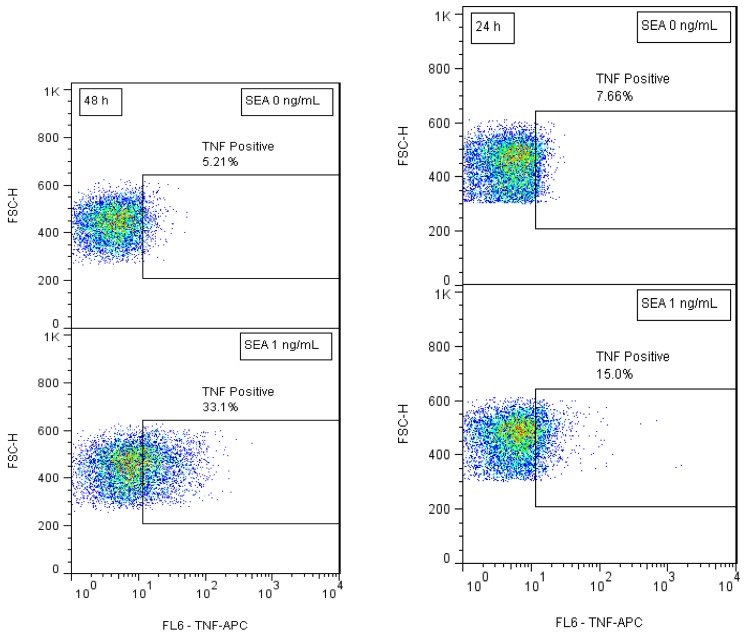
Intracellular TNF protein accumulation level within T cells for detection of biologically active SEA. CD4^+^ T-cells were stimulated with 1 ng/mL of SEA for 24 h and 48 h. Brefeldin A is added to prevent TNF secretion from the cells. The cells were fixed, permeabilized and stained with antibody against TNF. Flow cytometric analysis was used to measure TNF production by the cells.

Our preliminary results suggested that short term SEA stimulation of primary naïve CD4^+^ T-cells increases TNF expression. In order to develop biomarkers for early detection of active SEA we stimulated naïve CD4^+^ T-cells with SEA for 24 h and 48 h. After intracellular staining, kinetic TNF production was assessed by flow cytometry. Our flow cytometric analysis shows (Figure 2) that SEA exposure increases the percent of cells that expressed TNF. The data also show that the production of TNF increased over time and that SEA can be detected at a concentration of 1 ng/mL after 24 h.

### 3.3. Stimulation with SEA Induces Differential Protein Expression of TNF

**Figure 3.**
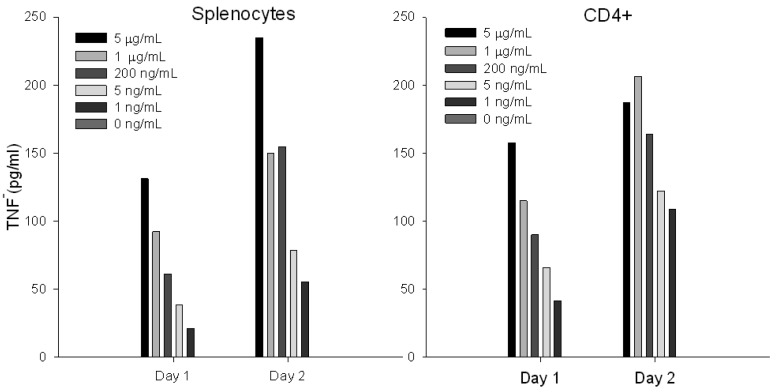
Flow cytometric analysis for quantification of biologically active SEA. Primary naïve CD4^+^ T-cells or splenocytes were spiked with increasing concentrations of SEA. After 24h or 48h of stimulation with SEA, TNF protein secretion was measured by flow cytometry.

To assess whether short term stimulation will induce differential secreted TNF following SEA treatment, we incubated primary naïve CD4^+^ T-cells or splenocytes with increasing concentrations of SEA. TNF protein secretion was analyzed on a FACSVantage SE Flow Cytometer. Our results (Figure 3) show that *ex vivo* SEA stimulation of primary naïve CD4^+^ T-cells or splenocytes induces TNF protein secretion in a dose dependent response after 24 h.

### 3.4. Early Detection of Biologically Active SEA in Food by ELISA

**Figure 4.**
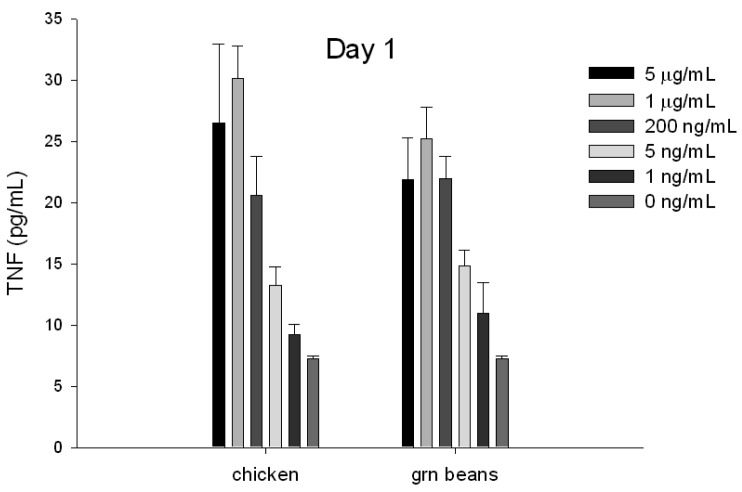
Quantification of biologically active SEA in food using ELISA assay. Chicken and green beans were spiked with increasing concentrations of SEA. Immunomagnetic beads were used to extract the toxin. Eluted toxin from beads was incubated with splenocytes. TNF secretion was measured by ELISA assay. Error bars represent standard error.

To evaluate if TNF protein secretion from splenocyte cells can be used for early detection and quantification of specific SEs in food, chicken and green beans were spiked with increasing concentrations of SEA. The toxin was extracted with immunomagnetic beads coated with anti-SEA antibody. The eluted toxin was incubated with splenocyte cells for 24 h. The ELISA data show (Figure 4) that toxin extracted from food can be detected after 24 h.

## 4. Discussion

In the present study, we evaluated a potential alternative method to the complex monkey or kitten bioassays for measuring biological activity of SEs. Here, we combined several approaches to detect and to quantify specific active SEs. We used SEA, which is associated with 75% of food borne staphylococcal outbreaks [13] and immunomagnetic beads coated with anti-SEA antibody to extract specific SEs from food. The cell proliferation assay, which measures incorporated BrdU into cellular DNA, is very sensitive with good reproducibility. However, the assay is time-consuming requiring several days of treatment with the toxin. In this work, we found that TNF represents an early molecular response or biomarker to SEA. TNF expression and secretion can be detected in less than 24 h. We used two different flow cytometric analyses for quantitative measurement of TNF: cytometric bead array that quantifies TNF protein secretion and quantitative intracellular TNF accumulation. To detect TNF protein accumulation within CD4^+^ T-cells, Brefeldin A was added to prevent TNF secretion. The cells were then fixed, permeabilized, stained with APC- conjugated anti-TNF antibody and analyzed by flow cytometry. Although our flow cytometric results were rapid and sensitive, using this analysis requires expensive equipment and skilled personnel. To overcome this aspect, we used an ELISA test that is relatively simple, inexpensive and practical for testing a large number of samples. The results demonstrated that the immunology techniques tested; bead array, intracellular TNF accumulation, and the ELISA test can detect active SEA with the same sensitivity as the cell proliferation assay. Induction of TNF secretion by SEA is 48h faster than by cell proliferation. By using three independent approaches to measure TNF, the cited data suggest that TNF is an early molecular response to SEA and is differentially expressed after a short term exposure. Therefore TNF can be used for early toxin detection in food.
